# Magnitude and determinants of plant root hydraulic redistribution: A global synthesis analysis

**DOI:** 10.3389/fpls.2022.918585

**Published:** 2022-07-22

**Authors:** Guisen Yang, Lei Huang, Yafei Shi

**Affiliations:** ^1^Shapotou Desert Research and Experiment Station, Northwest Institute of Eco-Environmental Resources, Chinese Academy of Sciences, Lanzhou, China; ^2^University of Chinese Academy of Sciences, Beijing, China

**Keywords:** plant roots, hydraulic redistribution, magnitude, influencing factors, terrestrial ecosystems, soil texture

## Abstract

Plant root hydraulic redistribution (HR) has been widely recognized as a phenomenon that helps alleviate vegetation drought stress. However, a systematic assessment of the magnitude of HR and its drivers at the global scale are lacking. We collected 37 peer-reviewed papers (comprising 47 research sites) published in 1900–2018 and comprehensively analyzed the magnitude of HR and its underlying factors. We used a weighting method to analyze HR magnitude and its effect on plant transpiration. Machine learning algorithms (boosted regression trees) and structural equation modeling were used to determine the influence of each factor on HR magnitude. We found that the magnitude of HR was 0.249 mm H_2_O d^−1^ (95% CI, 0.113–0.384) and its contribution to plant transpiration was 27.4% (3–79%). HR varied significantly among different terrestrial biomes and mainly occurred in forests with drier conditions, such as temperate forest ecosystems (HR = 0.502 mm H_2_O d^−1^), where HR was significantly higher than in other ecosystems (*p* < 0.01). The magnitude of HR in angiosperms was significantly higher than that in gymnosperms (*p* < 0.05). The mean magnitude of HR first increased and then decreased with an increase in humidity index; conversely, the mean magnitude of HR decreased with an increase in water table depth. HR was significantly positively correlated with root length and transpiration. Plant characteristics and environmental factors jointly accounted for 61.0% of the variation in HR, and plant transpiration was the major factor that directly influenced HR (43.1% relative importance; *p* < 0.001), and soil texture was an important indirect driver of HR. Our synthesis offers a comprehensive perspective of how plant characteristics and environmental factors influence HR magnitude.

## Introduction

Hydraulic redistribution (HR) is the passive movement of water from moist to dry soil through plant roots, including the lifting of water from the deeper to shallower soil layers (hydraulic lift, HL), the movement of shallow to deep soil layers (downward hydraulic redistribution, DHR), and lateral transportation (Burgess et al., 1998; Neumann and Cardon, 2012). HR generally occurs in the root-soil interlaced area, where exchanges of mass and energy are the most frequent and active in the soil ecosystem (Leffler et al., 2005; Prieto et al., 2012). The phenomenon is present worldwide within a range of different ecosystems and plant species (Bogie et al., 2018). HR can effectively increase the water content of dry soil, support the vigor and conductivity of fine roots, improve microbial activity, and promote nutrient absorption (Lee et al., 2018; Wang et al., 2018). Early research mainly focused on arid and semi-arid areas, and then gradually expanded to seasonally arid and humid and semi-humid areas (Jackson et al., 2000; Pomazkina and Lubnina, 2002). The latest research reported that HR has been observed in more than 120 plant species from different bioclimatic zones (Fu et al., 2018). HR may affect the net primary productivity of plant communities, vegetation distribution patterns, biogeochemical cycles, and ultimately affect climate (Sun et al., 2018).

Although the amount of water transported through HR is extremely low compared to precipitation, it is increasingly recognized as an important because of its substantial role in the effective water use of plants (Neumann and Cardon, 2012; Lee et al., 2018), such as supporting plant life activities (Fu et al., 2018), prolonging the lifespan of plant fine roots (Meinzer et al., 2004), enhancing the activities of root hairs, and reducing root embolisms under drought stress. The reported average amount of HR varied with species and environmental conditions. For example, the contribution of HR to the upper 100 cm of soil was 0.7 mm H_2_O d^−1^ near the middle of the dry season, while in the wet season HR was exceptionally low (Scholz et al., 2010). In addition, it was found that savanna trees in a campo cerrado released about 0.004 mm of water per day to the upper soil layers *via* HR, and was 0.008 mm per day in a denser savanna site (Scholz et al., 2008a). Empirical estimates of the average magnitude of upward HR span more than an of magnitude, from 0.04 mm H_2_O d^−1^ in a Brazilian savanna (Scholz et al., 2010) to 1.3 mm H_2_O d^−1^ in New England sugar maples (Emerman and Dawson, 1996). Neumann and Cardon (2012) synthesized that the magnitude of HR varied from 0.04 to 1.30 mm H_2_O d^−1^, and 0.1 to 3.23 mm H_2_O d^−1^ in the empirical and modeling studies. However, a more comprehensive and quantitative analysis of how HR varies across a range of different ecosystems and plant species is lacking.

Most of the studies that investigated HR and its relationship with environmental and biological factors were confined to field or plot scale, and so too were modeling studies (Yu et al., 2013). These studies focused on the magnitude of HR by individual plant species at specific sites and the possible drivers of HR (Bogie et al., 2018; Meunier et al., 2018). Several studies have reported that HR occurs only under certain conditions (Neumann and Cardon, 2012; Hafner et al., 2017). The occurrence and magnitude of HR differs among plant species, even between individuals of the same species under different environments (Neumann and Cardon, 2012). Factors that affect HR include climate (precipitation and evapotranspiration), soil characteristics (soil moisture, soil texture, and land use type; Hafner et al., 2020, and vegetation characteristics (morphological characteristics and distribution of roots, root length, and root water storage ability; Leffler et al., 2005). Although these studies provided an abundance of information at a local scale, they provided little information about the general patterns of HR production at larger spatial scales (Nadezhdina et al., 2015; Yu and D'Odorico, 2015). Moreover, it is difficult to extrapolate these results between sites, and very few studies have attempted to explain the differences in HR magnitude on a regional scale. Therefore, what is the spatial pattern of the magnitude of HR on a global scale? What factors determine the magnitude of HR and how do they affect HR? Understanding these processes could facilitate the evaluation of the significance and effectiveness of HR in plant water use in terrestrial ecosystems. In addition, the quantification of HR could also provide basic data for global groundwater resource assessment and modeling (Zhang and Zwiazek, 2018), determining vegetation water use efficiency and modeling, and other hydrological and surface models (Wang et al., 2018).

Here, we aimed to explore the global patterns of HR to determine which factors are most influential in HR magnitude and to systematically evaluate the responses of HR to its drivers. To accomplish these aims, we compiled a global dataset of 47 HR observations of terrestrial plants (e.g., trees, shrubs, and herbs) extracted from 37 papers published between 1900 and 2018 (Supplementary Data Sheet 1). We used a weighting method to determine the mean magnitude of HR and its contribution to plant transpiration. We used machine learning (boosted regression trees) and structural equation modeling to analyze the influence of each factor on HR quantity.

## Materials and methods

### Literature search and data compilation

Through the Web of Science, we searched for published literature under “plant root hydraulic redistribution”. We found 400 pieces of literature from 56 countries published between 1900 and 2018. Through title and abstract screening, we excluded papers that only provided qualitative descriptions of HR but did not clearly measure the specific magnitude of HR (DeMalach et al., 2017). In total, 37 papers were identified for integrated analysis, which met our requirements for data extraction, and included 47 research sites and 21 species of plants across five biomes. We also extracted other relevant information in the study, such as soil type, plant root length, sampling date, regional precipitation, evaporation, water table depth, biome type, latitude, longitude, and climate variables. These observations accounted for the amount of HR for different plant species at a specific site, accounting for the proportion of plant transpiration, and its effect on ecology and hydrology. Furthermore, we calculated the average values of HR (M), sample size (n), standard deviation (SD), and 95% confidence intervals (CI) for the corresponding average HR values. We found that HR research included two common methods: field measurement and model simulation. Since we were interested in the magnitude of HR, we treated independent research conducted by different institutions or researchers at the same location as different research results, including research plots where some field measurements and model studies overlapped. We collected 47 research sites (field studies and model studies, shown in Supplementary Figure 1).

We adopted the following four criteria to select suitable studies:

(1) The magnitude of HR and its influencing factors were determined through field or modeling studies.
(2) The amount of HR by a specific plant species was measured.
(3) The average value and standard deviation of HR could be directly obtained from the literature or could be calculated indirectly.
(4) Papers in which filed observations occurred during less than a full growing season were excluded.

We extracted the following explanatory variables for each study:

(1) Location (latitude and longitude)—in cases where the studies did not report the latitude or longitude (5% of study sites), the approximate latitude and longitude were derived by geocoding the name of the location in Google Earth.
(2) To conduct biome-level analysis, we aggregated the data into seven biomes based on the definitions of the International Geosphere-Biosphere Programme (IGBP): needleleaf or broadleaf forest, temperate forest, deserts or sparsely vegetated, temperate grassland, savannahs, barren land, and shrublands (Supplementary Figure 2; Xu et al., 2013).
(3) Humidity index is the ratio of mean annual precipitation to mean annual evapotranspiration. In cases where the original source studies did not report precipitation or evapotranspiration, it was extracted from WorldClim version 2.1 using the site's geographic location (i.e., latitude and longitude).
(4) Water table depth (m)—for literature that did not report groundwater level information in the study area, the water table depths were extracted from the global patterns of the groundwater table depth dataset (Fan et al., 2013).
(5) Plant root length (cm)—the midpoint length of plant roots were calculated as the root length variable for analysis.

### Measurement methods of HR from original literature

#### Field measurement

Field measurements were mainly conducted using either of two methods, and the HR unit obtained by the two methods is unified into water volume (mm H_2_O d^−1^). The soil moisture method divides the plant roots into upper and lower or left and right zones. The circulation of soil moisture in the two zones occurs by means of soil infiltration. One zone is provided with sufficient water supply, and the other zone is deprived of water. HR is estimated by measuring the changes of soil water content (θ) and soil water potential (ϕ) in the arid zone. The θ declines during the day when plant and root demand were highest. At night, HR moves water *via* roots from wetter soil layers to drier soil layers following a water potential gradient. This night-time increase in θ in the absence of precipitation is considered to be HR. The magnitude of daily HR was estimated for each sensor and then integrated across the profile to provide total daily HR within the monitored soil layer (Brooks et al., 2002; Warren et al., 2007; Cleverly et al., 2016).


(1) $$\begin{array}{r} HR=\emptyset_{\max}\left(day\;\left(x+1\right)\right)-\emptyset_{\min}\left(day\;x\right) \end{array}$$

Soil water potentials (ϕ) were usually quantified using thermocouple psychrometers (PST-55, Wescor, Logan, UT) installed soil layers at different depths. The θ was quantified using multi-sensor, frequency domain capacitance probes. A statistical program (Sigma Plot 7.101, SPSS Inc., Chicago, IL) was used to fit a simple three-parameter non-linear regression curve to the data at each depth (Warren et al., 2005):


(2) $$\begin{array}{r} \varphi =\frac{-1}{{\left(a+b\theta \right)}^{c}} \end{array}$$

where *a, b, c* are parameters determined by the regression.


(3) $$\begin{array}{r} \varphi =\varphi_{cr}{\left(\frac{\theta_{r}-\theta }{\theta_{r}-\theta_{s}}\right)}^{\frac{1}{\lambda }} \end{array}$$

where θ_*s*_ is the saturated soil volumetric water content, θ_*r*_ is the residual soil volumetric water content (for very dry soil), ϕ_*cr*_ is the soil water potential as u approaches saturation, and λ is a parameter related to soil porosity.

The sap flow method involves installing heating and control probes for measuring stem flow on the lateral roots and main roots of plants, respectively. By measuring the temperature difference between the heating probe and the control probe, the liquid flow velocity is calculated, and the total liquid flow per unit time is calculated. The commonly used determination methods include the thermal ratio method (HRM), thermal field deformation method (HFD), and thermal diffusion technology (TDT). Taking the HRM method as an example, several sets of sensors are placed in the stem and taproot and single sets of sensors are placed in major lateral roots. In addition, the cross-sectional area of the monitored lateral root is extracted. For each species, mean and standard deviation (SD) sap velocity were calculated for all lateral root measurements, and all tap root measurements and these values were then multiplied by the total cross-sectional area of roots in each class of root. HR was quantified as the total volume of water estimated from negative sap flow (i.e., flow directionally away from the trunk) measured on roots. HR was presented as night-time sap flow (g/day) by summing the product of sap velocity by the cross-sectional area of similar lateral roots and water density, instead of volumetric flow velocities (mm/day) because it is difficult to scale up with the size of each individual lateral root monitored. It is difficult to measure sap flow on all roots, so only large lateral roots are instrumented with sap flow sensors (Burgess et al., 2001; Yu et al., 2018).

In addition, the isotope tracer method has also been widely used to study HR, but we did not include them in our analysis because stable isotope tracer technology is mainly used to discover the occurrence of HR, and it is difficult to accurately measure the amount of HR. Finally, the quantity unit of HR obtained by the two methods is unified into water volume (mm H_2_O d^−1^) using this method.

#### Model simulation

In addition to field measurements, models can be used to estimate quantify HR. The overall goal of such a model is to capture the influence of soil water content on HR dynamics and magnitude according to the conductivity of soil, soil roots and roots. The original HR model was posited by Ryel et al. (2002), and is now widely used and known as the “Ryel model.” This model also laid a foundation for the establishment of other models in HR research.

Water movement among soil layers by roots has been assumed to occur based on differences in Ψ_*i*_, with water moving from wetter to drier layers (Caldwell et al., 1998). Water redistributed by roots was modeled as a function of the distribution of active roots, radial conductivity of water between the root-soil interface (rhizosphere conductance), and transpiration activity (Ryel et al., 2002). The Ryel model defines HR as a function of hydraulic conductivity in root water flow path and water potential gradient in different soil layers, and HR (HR*i*) of a certain soil layer *i* can be expressed as follows:


(4) $$HR_{i}=C_{\max}\sum_{j}\left(j-1\right)\max(c_{i}-c_{j})R_{ij}D_{tran}$$

where *C*_max_ is reduced using an empirical relationship from van Genuchten (range 0–1) as soil water potential Ψ decreases (i.e., soil dries) in the source (*c*_*j*_) or the sink (*c*_*i*_) soil layers. Conductance is distributed among soil layers as a function (*R*_*ij*_) of root biomass distribution in the layers. Because this approach does not model flow within the root system itself, and therefore does not simulate root water potential, it cannot easily capture the competition for xylem water between atmospheric water demand (*via* transpiration) and dry soil layers. Ryel et al. (2002) therefore included an “on /off” term, *D*_*tran*_, that restricts redistribution to periods with low transpiration demand. For example, Zheng and Wang (2007); Baker et al. (2008), and Wang (2011) adopted Ryel et al.'s (2002) formulation. Scholz et al. (2010) slightly altered the effective conductance calculation to focus on the drying (water-receiving) soil layer's control over flow. Other models are also used to study HR, such as the big root model (Amenu and Kumar, 2008), macro–meso scale models (Siqueira et al., 2008), and the dynamic root profile model (Schymanski et al., 2008).

### Transpiration of plants

Leaf transpiration losses from the soil were assumed to be primarily limited by the soil-root conductance for water in each layer. The transpiration rate was further limited by the portion of roots within each layer, the sap flow of stems and roots using the heat ratio method (Burgess et al., 2001). Whole-tree crown-related sap flow (equal to transpiration, mm/hr) was calculated by dividing the product sap velocity (cm/hr) and sapwood area (cm^2^) by the crown area (cm^2^). The crown area was calculated as the circular area *via* measurement of diameter of crown in four directions. Then, the daily transpiration of plants was converted into a unit (mm H_2_O d^−1^) consistent with the HR (Yu et al., 2018).

### Effect size of HR

Confidence intervals (CI) indicate the range within which the true mean (the magnitude of HR) estimates fall in 95% of all possible integrated analyzes. The 95% CI was computed using the following equation (Evaristo and Mcdonnell, 2017):


(5) $$\begin{array}{r} \text{Lower limit}=M-1.96^{*}\frac{\text{SD}}{\sqrt{n}} \end{array}$$

(6) $$\begin{array}{r} \text{Upper limit}=M+1.96^{*}\frac{\text{SD}}{\sqrt{n}} \end{array}$$

where M is the average value of HR, SD is the standard deviation corresponding to the HR value, and *n* is the sample size in each study.

The ultimate goal of any integrated analysis is to provide a cross-site comparison and an overall view or effect size (in this case, the magnitude of HR; Zhang et al., 2021). If the precision across all 37 published papers in our database was equal, we could readily compute the simple mean of all HR estimates. As this was not the case, we needed to compute a weighted mean by assigning weights to the studies. Here, we weighted each study by the inverse of its original (within-study) variance. The weights (W_*i*_) allocated to each of the studies are then inversely proportional to the square of the standard deviation (SD) for the *i*-th study. This allocates greater weight to studies with smaller standard deviation (Gao and Yohay, 2020). Therefore, the weight calculation formula we used was as follows:


(7) $$\begin{array}{r} W_{i}=\frac{1}{\text{S}{\text{D}}^{2}} \end{array}$$

### Data and statistical analysis

We used boosted regression trees (BRT) analysis to estimate the effects of individual predictor variables on HR. Plant transpiration and plant root length were combined as plant characteristics and water table depth, humidity index, mean annual precipitation, and mean annual evapotranspiration metrics were combined to the environmental factors. BRTs are robust to collinearity between variables, variable outliers, and missing data, which is thought to be advantageous in this study as there are many category predictors and little prior information. In addition, BRT has performed well in determining the important independent variables. We performed BRT analysis using the “gbm.step” function in the R “dismo” package (Zhang et al., 2021).

We used GraphPad Prism 6 (GraphPad Software Inc., San Diego, CA, USA) software to complete the HR effect size graphic plotting. The differences of HR in different classifications were measured by one-way analysis of variance (ANOVA) and an independent-sample Kruskal-Wallis test was used to test for differences in HR between soil textures, which were performed using SPSS 22.0 (IBM Corp., Armonk, NY, USA). We created a map using ArcMap 10.6 in ArcGIS 10 (ESRI, Redlands, CA, USA). The fitting curve of the influencing factors was completed using Origin 9.1 (OriginLab Corp., Northampton, MA, USA). We used machine learning (boosted regression tree) to analyze the influence of the individual variables on HR (Zhang et al., 2021). A mixed-model structural equation model (SEM) was constructed using AMOS 21.0 to determine how the magnitude of HR was driven by plant characteristics and environmental factors. Before modeling, we first considered a full model that included all possible environmental factors, and pathways, and eliminated non-significant ones. To test the overall goodness of fit for the SEMs, we used the χ^2^ test and the root mean square error of approximation (RMSEA; García-Palacios et al., 2015).

## Results

### Magnitude of HR and its contribution to plant transpiration demands

**Figure 1:**
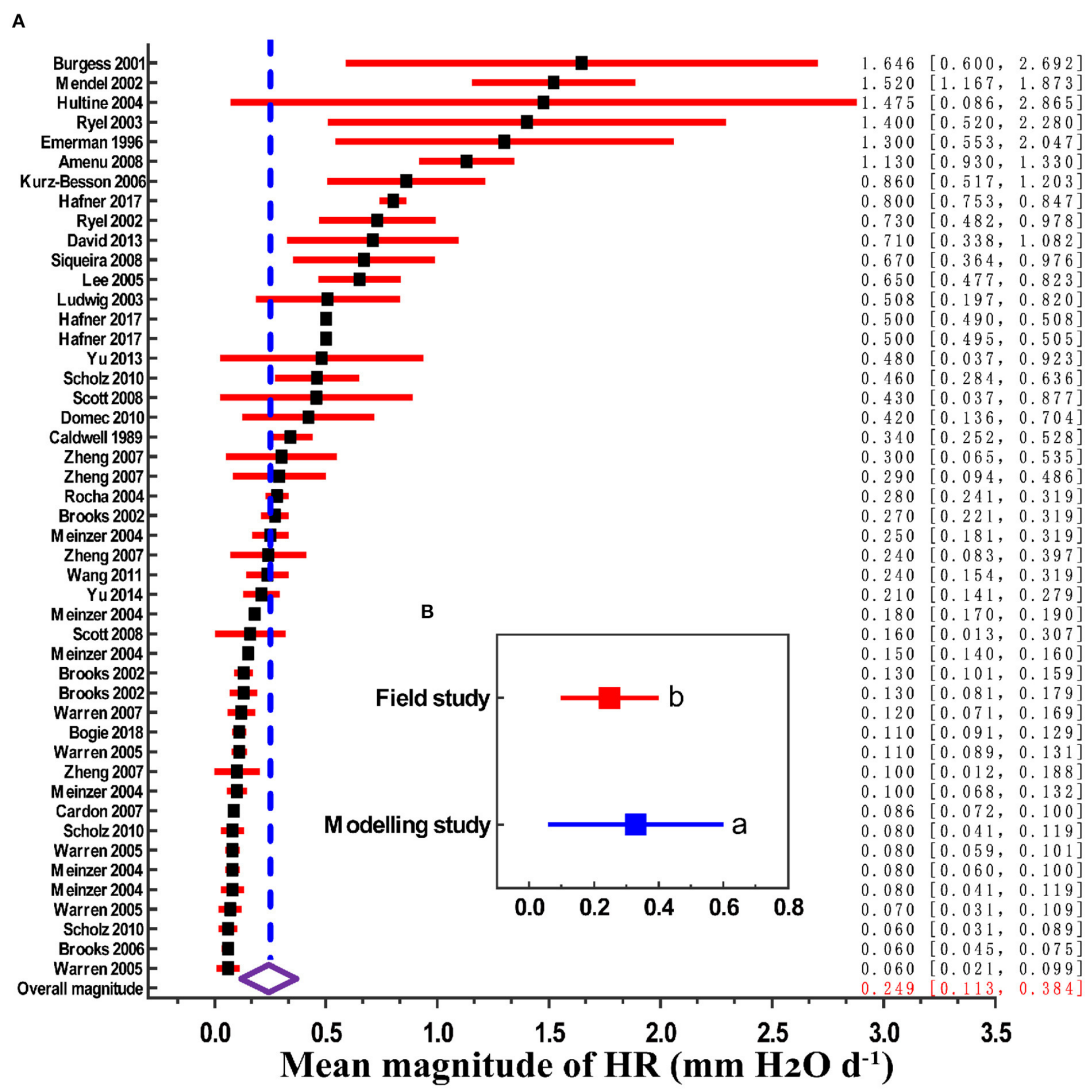
Mean magnitude of HR (x-axis: 0 corresponds to no HR; > 0 corresponds to the actual measured mean amount of HR). **(A)** Magnitude estimates grouped by source paper (first author-year format). Filled black squares are magnitude point estimates, error bars are 95% CI (red horizontal lines). The open diamond represents the overall magnitude value, and its 95% CI is represented by the width of the diamond. **(B)** Magnitude estimates grouped by field study and modeling study; different lowercase letters indicate significant difference at the 0.05 level.

We found that the global estimated magnitude of HR was 0.249 mm H_2_O d^−1^ (95% CI, 0.113–0.384). However, there is considerable range in HR between studies, from 0.06 mm H_2_O d^−1^ in a Brazilian savannah to 1.646 mm H_2_O d^−1^ in Western Australia eucalyptus, which reflected site and species-level differences across studies (Figure 1A). In addition, the mean magnitude of HR among the modeling studies was 0.319 mm H_2_O d^−1^ (*n* = 12), and for the field studies it the mean magnitude was 0.248 mm H_2_O d^−1^ (*n* = 35). The modeling results were significantly higher than those of field measurements (Figure 1B, *p* < 0.01).

**Figure 2:**
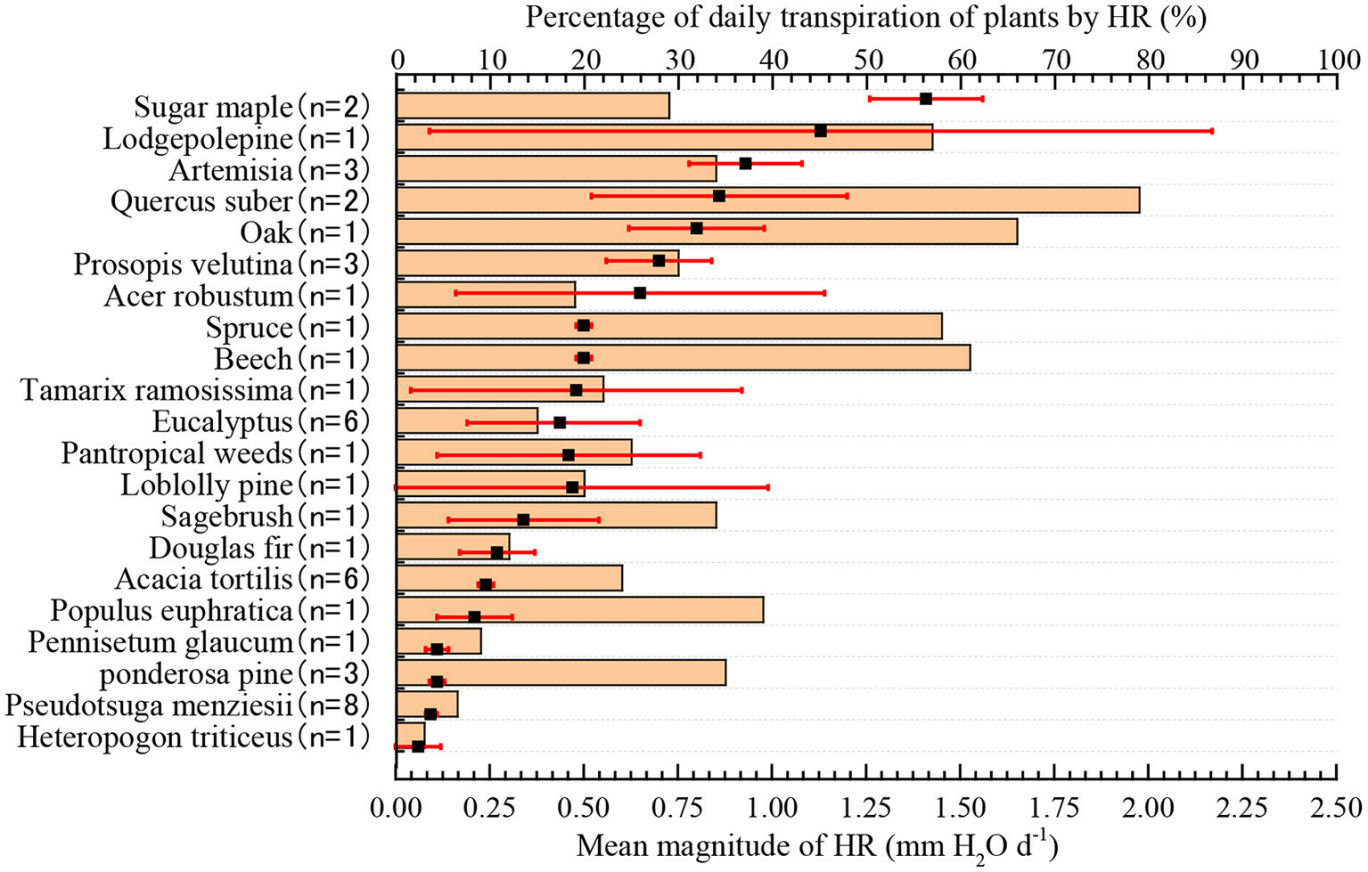
The amount of HR and its percentage of average daily transpiration. The filled black square represents the mean plant HR, and the red error bar is a 95% CI. The tan bars represent the percentage of HR in the average daily transpiration of plants.

The amount of HR in different plant species that accounted for the proportion of daily transpiration of plants was significantly different, ranging from 3 to 79%, with an average value of 27.4% (Figure 2, *n* = 47). Compared with shrubs and trees, the magnitude of HR had a lower influence on plant transpiration in herbs. For example, the smallest influence of the magnitude of HR was observed in *Heteropogon contortus*, in which HR accounted for only 3% of the variation in transpiration. Conversely, in *Quercus robustus*, the largest species, the average daily HR volume accounted for 79% of the daily transpiration volume, which played a very important role in relieving water stress in the dry season and maintaining healthy growth.

### Magnitude of HR in different terrestrial biomes

**Figure 3:**
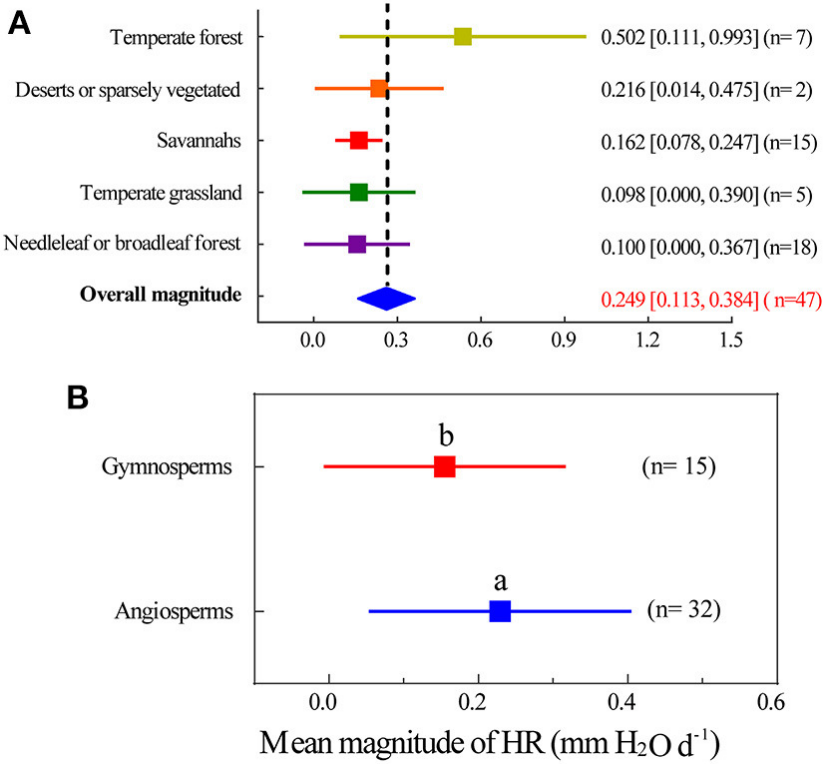
Mean magnitude of HR of different vegetation types. **(A)** Magnitude estimates grouped by different terrestrial biomes. **(B)** Magnitude estimates grouped by angiosperm and gymnosperm. Filled squares are magnitude point estimates. Error bars are 95% CI. The solid diamond represents the average magnitude of HR and its 95% CI is represented by the width of the diamond. Different lowercase letters indicate significant difference at the 0.05 level.

The HRs at biome and global scales are summarized in Appendix S1. Most of the field sites were in located in North America, South America, and Europe. There were fewer observations for Africa, Russia, Asia, and Antarctica. On a terrestrial biome basis (Figure 3A), HR had the greatest magnitude in temperate forests 0.502 mm H_2_O d^−1^ (95% CI, 0.111–0.993) and deserts or sparsely vegetated land 0.216 mm H_2_O d^−1^ (95% CI, 0.014–0.475). HR had the smallest magnitude in needleleaf and broadleaf forest 0.100 mm H_2_O d^−1^ (95% CI, 0–0.367), temperate grassland 0.098 mm H_2_O d^−1^ (95% CI, 0–0.390), and savannahs 0.162 mm H_2_O d^−1^ (95% CI, 0.078–0.247). In addition, angiosperms exhibited a greater magnitude of HR at 0.281 mm H_2_O d^−1^ (95% CI, 0.053–0.405) than gymnosperms at 0.102 mm H_2_O d^−1^ (95% CI, 0–0.323), and this difference was significant (Figure 3B, *p* < 0.01).

### Factors influencing HR

**Figure 4:**
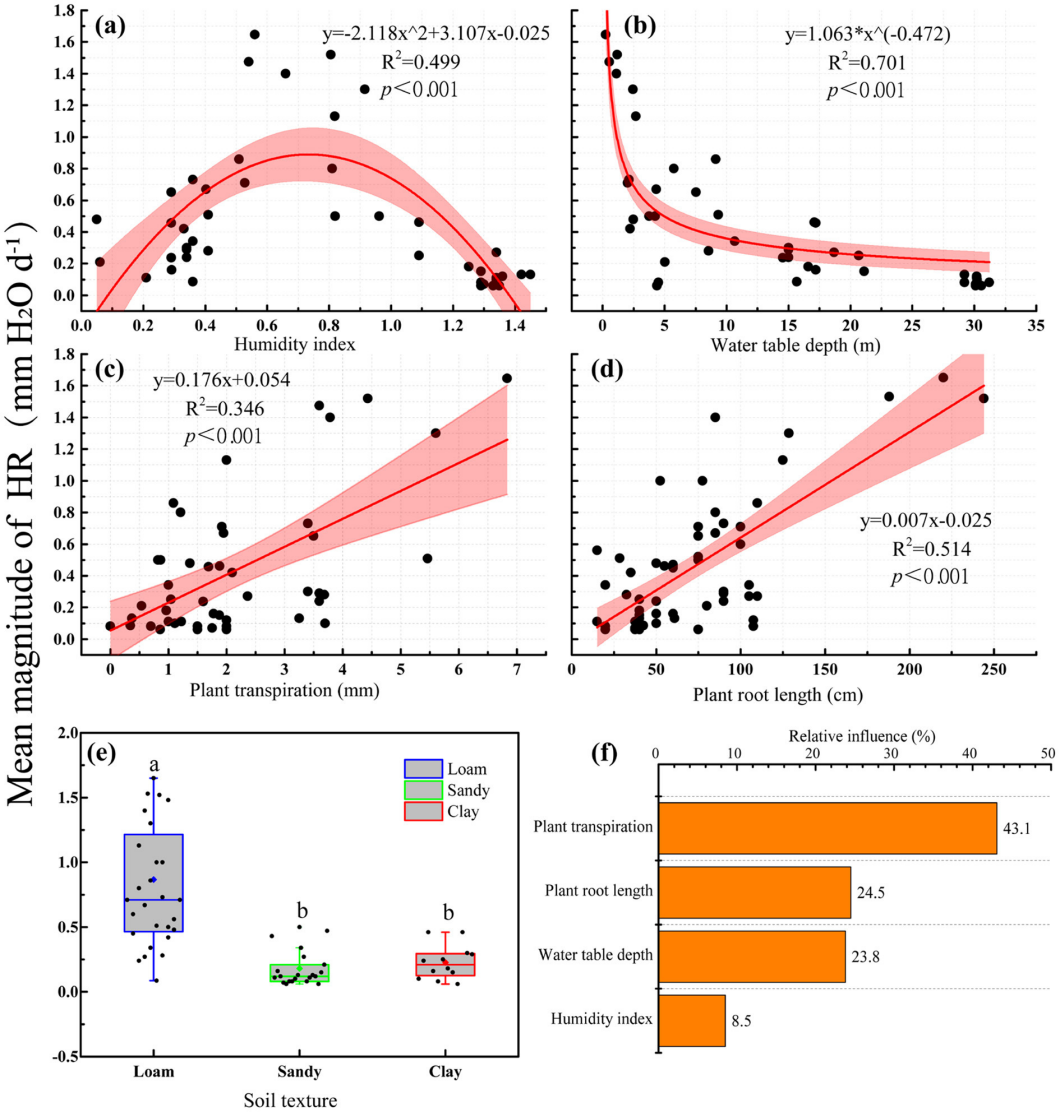
The factors influencing HR. Relationship between HR and environmental factors **(a–d)**; **(e)** influence of soil texture on HR. The boxplots characterize the lower, median, upper quartiles, and the interquartile range (upper quartile–lower quartile), which covers the central 50% of the data. The whiskers represent 95% of the data. The diamond within each boxplot represents the mean and each small circle represents one individual observation. **(f)** Relative percentages of the influence of vegetation characteristics and environmental factors on HR magnitude. Different lowercase letters indicate significant difference at the 0.05 level.

With an increased humidity index, the amount of HR first increased and then decreased (Figure 4a, *p* < 0.001, *R*^2^ = 0.499). The amount of HR decreased as water table depths increased (Figure 4b, *p* < 0.001, *R*^2^ = 0.701), but increased with an increase in plant transpiration (Figure 4c, *p* < 0.001, *R*^2^ = 0.346) and soil-plant root length (Figure 4d, *p* < 0.001, *R*^2^ = 0.514). We ranked the driving factors using the boosted regression trees. We found that plant transpiration was the major factor influencing HR (relative importance was 43.1 %), followed by plant root length (24.5%), water table depth (23.8%), and humidity index (8.5%; Figure 4f). In addition, we used an ANOVA to compare the relationship between HR and soil texture, and it was found that HR was significantly higher in loam than in sandy soil and clay (Figure 4e, *p* < 0.001).

**Figure 5:**
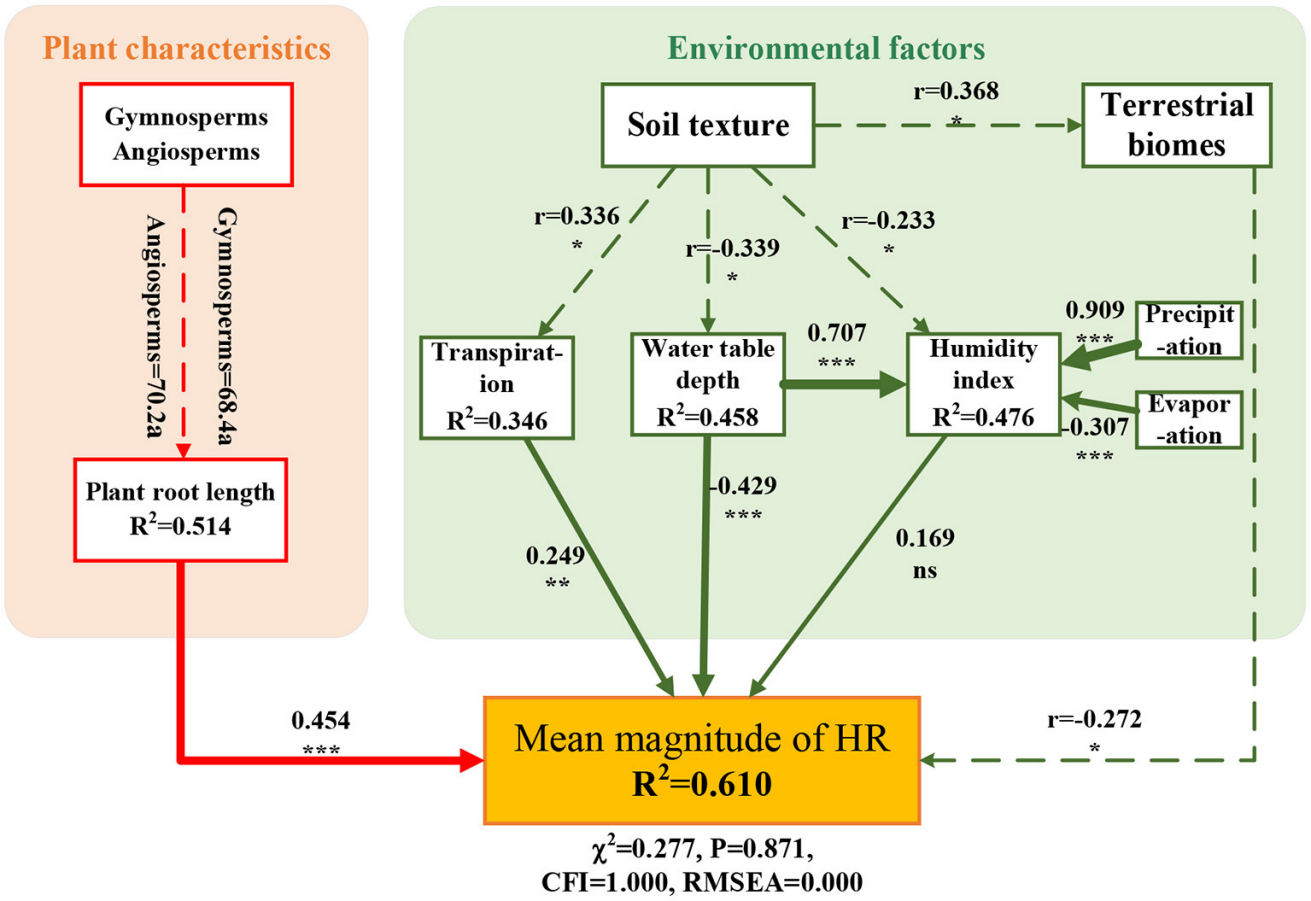
Direct and indirect effects of environmental factors on the hydraulic redistribution (HR) magnitude based on structural equation modeling (SEM). SEM fitted with range-standardized coefficients, which link plant type, root length, soil texture, terrestrial biomes, transpiration, water table, and humidity index on the magnitude of HR (χ^2^ = 0.277, *P* = 0.871, CFI = 1.000, RMSEA = 0.000). The number next to the solid arrow represents the normalization coefficient, and the line width corresponds to the strength of the standardized coefficient. Notably, gymnosperms, angiosperms, soil texture, and terrestrial biomes are non-numerical variables. The dashed line shows the correlation between variables, and r is the correlation coefficient. Letters a denote groupings based on *post-hoc* tests. Significance levels are as follows: ***p < 0.001, **p < 0.01, *p < 0.05, and ns indicates not significant.

The SEM linking the mean magnitude of HR with both plant characteristics (e.g., plant seed species and root length) and environmental factors (e.g., water table depth, humidity index, and average transpiration) as predictors had a good fit to the data and accounted for 61.0% of the variation in HR (Figure 5, χ^2^ = 0.277, CFI = 1.000, *P* = 0.871, RMSEA = 0.000). Soil texture had an important indirect effect on HR. Although the difference in root length between angiosperms and gymnosperms was not significant, their effect on HR was significant. In addition, soil texture was highly correlated with plant transpiration, water table depth, and terrestrial biome. These factors jointly affected the magnitude of HR in plant roots.

## Discussion

### The magnitude of HR and its contribution to plant transpiration water demand

Our study summarized the mean magnitude of HR at the global scale, which is an important step toward a better understanding of regional variation in the magnitude of HR (Zhou et al., 2020; Sian and Menge, 2021). We found that the global estimated magnitude of HR was 0.249 mm H_2_O d^−1^, which is relatively lower than reported by Neumann and Cardon (2012), who found that the mean magnitude of HR was 0.3 mm H_2_O d^−1^. The reason for this difference may be that we used the weighted average method to calculate the magnitude of HR, but this method can more accurately evaluate the errors caused by the difference of sample size in different studies (Veroniki et al., 2016). In addition, the samples collected in our study were larger. Previous studies of HR were either oriented to specific regions or paid little attention to cross-site influence factors (Prieto et al., 2012; Meunier et al., 2018; Zhang and Zwiazek, 2018). However, our dataset was more comprehensive, and our research results can better represent the actual occurrence of HR. We also found that the magnitude of HR varied greatly depending upon the ecosystem types (Figure 3A). For example, temperate forests were significantly higher than the other ecosystem types, possibly because this area is mainly distributed in humid and sub-humid areas with seasonal droughts (Scholz et al., 2010), which is more conducive to HR occurrence (Neumann and Cardon, 2012). In addition, compared with tropical regions and temperate grasslands, the amount of HR in deserts was also higher. This finding was consistent with the traditional perspective that dry environments are one of the necessary conditions under which to HR occurs (Caldwell et al., 1998; Horton and Hart, 1998). These biome-level estimates of mean magnitude reflect the variability between major habitat types and underline differences across study sites and species (Evaristo and Mcdonnell, 2017).

Another major finding was that HR in the modeling studies were significantly higher than what was found from the field measurements (Figure 1B). The main reason for this difference could be the sensitivity of modeled HR to root and root-soil conductance (Neumann and Cardon, 2012). The model study quantified (parameterized) HR as a function of water potential between different soil layers (Amenu and Kumar, 2008). Previous studies have reported that the amount of HR is directly proportional to the radial soil-root conductance (Mendel et al., 2002; Wang, 2011). For example, Mendel et al. (2002) found that over the tested range, with each of magnitude increase in radial conductivity of rootlets, HR increased by a factor of 1.4. Thus, this relationship also be a reason for the uncertainty in the HR magnitude research (Zheng and Wang, 2007). Another reason was that the premise of model simulation research on HR is that the stomata of plants are open during the day and completely closed at night, so the inhibition of transpiration of plants at night on HR was ignored in model research (Dawson et al., 2007). In our study, we used 12 model simulation cases and the weighted average method to further confirm the findings of Siqueira et al. (2008) and Wang (2011). In addition, the amount of HR of angiosperms was significantly higher than that of gymnosperms (Figure 3B). This difference may be because the main conducting elements in angiosperms (xylem vessels) allow for wider variability in element size and wall thicknesses than their conducting element counterparts in gymnosperms (tracheids; Anderegg, 2015). Furthermore, angiosperms have a greater number of parenchyma cells, which are linked to improved hydraulic system efficiency after stressful conditions such as drought (McDowell, 2011). Anatomical differences in the xylem of the two types of plants may explain our finding that angiosperms tend to be more favorable to HR than gymnosperms.

We found that HR accounted for 27.4% of the daily plant transpiration (Figure 2). In fact, this proportion was a very considerable of magnitude. We consider the magnitude of HR *per se* may be important, however, the proportion of its contribution to plant transpiration would more adequately reflect the eco-hydrological effects of HR (Sun et al., 2018). If HR contributes a portion of the water required to meet transpiration requirements, the direct hydrologic effect of HR may be significant. Conversely, if HR contributes only a small proportion of transpiration water, it may not have direct and significant hydrologic effects (Neumann and Cardon, 2012). If HR contributes to the transpiration of plants under long-term drought conditions, then HR will have far-reaching significance in promoting ecosystem productivity and protecting plants from drought stress. Previous studies have shown that transpiration increases by 10–40% because of HR in tropical systems (da Rocha et al., 2004), 20–25% in dry and arid environments (Bleby et al., 2010), 19–40% in mesic forests (Jackson et al., 2000), and up to 81% in some Mediterranean ecosystems (Kurz-Besson et al., 2006). However, most of the data provided by such studies are maxima and minima, which do not reliably reflect the average contributions of HR to plant transpiration water requirements. Our study used a weighted average algorithm to address the knowledge gap. In addition, we observed that the proportions of the influence of HR were relatively high in tree and shrub transpiration and low in herb transpiration. This is probably because herbs had shallow roots and were mainly distributed in tropical and subtropical regions where soil water is abundant.

### Analysis on influencing factors of HR

Overall, our synthesis provided a global assessment of how plant characteristics and environmental variables affect HR, which promotes a more comprehensive understanding of the mechanisms of plant root HR (Prieto et al., 2012). We found no significant correlation between annual precipitation and the magnitude of HR. This finding is consistent with the results of previous studies that reported a similar average HR with a mean annual precipitation ranged that from 550 to 2,500 mm (Meinzer et al., 2004). In this case, precipitation intervals and depth may be the key impact factors, which can trigger a cascade of plant physiological responses at different time scales (Huang and Zhang, 2016). However, the amount of HR was significantly correlated with the humidity index (precipitation/evaporation), and as humidity index increased, the amount of HR first increased and then decreased (Figure 4a), which suggested that HR reached an optimal condition when the ratio of precipitation to evaporation reached a certain condition (humidity index = 0.752). In contrast, an extremely dry or humid soil environment was not conducive to the occurrence of HR (Domec et al., 2004). Therefore, after plants have experienced a certain severity or period of drought, the strong transpiration causes the soil moisture to reach a certain level, which stimulates the occurrence of HR and makes it reach a maximum under suitable conditions (Neumann and Cardon, 2012).

The influence of plant root characteristics on the amount of HR has been widely confirmed, and research in this area has focused on the physiological and structural characteristics of plant roots, such as the distribution and pattern of roots or whether the roots have the function of releasing and absorbing water (Scholz et al., 2008b; Wang, 2011). We found that the average root length of plants was significantly positively correlated with the amount of HR (Figure 4d). This finding indicated that when the root length of the plant was longer, the HR was higher than that of plants with shorter roots. Since the heterogeneity of soil moisture content increases with the increase in soil depth, plant root systems must bridge a soil water potential gradient large enough to drive flow. For example, annual herb plants have shorter roots, the difference in shallow soil moisture is smaller, and the magnitude and range of HR are also smaller (Neumann and Cardon, 2012). On the other hand, the long root system of plants is conducive to the absorption and utilization of water sources other than soil moisture, such as river water and deep groundwater, which may increase the amount of HR (Rewald et al., 2015).

Soil texture can influence the potential magnitude of HR (Scholz et al., 2008b; Prieto et al., 2012). Our results demonstrated that the magnitude of HR in loam was significantly higher than in sand or clay (Figure 4e). This may be because the sand content, soil particle size, permeability, and water retention in loam are between sand and clay, which is more conducive to the occurrence of HR (Schymanski et al., 2008). In addition, soil texture affects HR by influencing the soil electrical conductivity and soil moisture. A series of empirical studies showed that with the drying of topsoil, HR first increased to its maximum value, then decreased or remained stable, which suggested that soil texture was important for the maintenance of HR (Warren et al., 2005; Scholz et al., 2008b; Prieto et al., 2010). This phenomenon has also been confirmed in the model simulation (Prieto et al., 2010). The reason may be that in soil with coarse texture, the root-soil contact (electrical conductivity) was more difficult to maintain as a larger aerated pore space would form (Schroder et al., 2008; Schymanski et al., 2008; Prieto et al., 2010).

We found that the amount of HR was negatively correlated with the depth of groundwater, and that the amount of HR decreased as the depth of groundwater increased (Figure 4b). This relationship may be because most of the lateral roots of plants are distributed in the shallow soil layers (Scholz et al., 2008b). A shallower groundwater level is beneficial for increasing the contact area between plant roots and groundwater, thus promoting the amount of HR (Neumann and Cardon, 2012). Studies have shown that New England sugar maple trees maintain a high amount of HR, mainly because the roots of sugar maple trees can reach groundwater (Emerman and Dawson, 1996). Groundwater will increase HR by 0.2 mm H_2_Od^−1^ during a simulated drought of up to 100 days in a stand of *Artemisia tridentate* (Ryel et al., 2002). Particularly in an ecosystem where the soil type is sandy, due to the poor water holding capacity of said soil, the groundwater source provides a sufficient water source for plant HR under drought stress (Neumann and Cardon, 2012). Results showed that increased plant transpiration enhanced the magnitude of HR (Figures 4C,F). Interestingly, in contrast to the above factors that influence HR, plant transpiration is the most significant driver. This is not coincident with Williams et al. (1993), who proposed that low plant transpiration can drive HR during rainstorms. The reason for this explanation is that previous studies focused on comparing the effect of plant transpiration on the amount of HR in certain environments, and thus could not evaluate the impact of plant transpiration on HR in different research sites or plant species (Hafner et al., 2020). Our works provide an insight into the relationship between plant transpiration and the amount of HR in different regions and for different plant species.

## Conclusion

In our synthesis, we determined the magnitude of HR and its contribution to plant transpiration demand and provided a global estimate. The mean magnitude of HR was 0.249 mm H_2_O d^−1^, which accounted for 24.7% of the daily plant transpiration. There were differences in the magnitudes of HR in different biomes. The magnitude of HR in temperate forests was significantly higher than in the other ecosystems. Plant characteristics and environmental factors jointly accounted for 61.0% of the variation in HR. Plant transpiration was the major driver of HR, and we found that soil texture played a key but indirect role in HR. Our study provided new knowledge on the global estimated magnitude of HR and how plant characteristics and environmental factors influence HR magnitude. Further research on HR should focus on the possible synergistic, additive, or antagonistic effects of multiple factors, which will require more empirical studies of multiple factors to clarify the combined effects.

## Data availability statement

The authors declare that the data associated with this study are provided as the Supporting Information Data S1 and R scripts, which are provided *via* Dryad (https://datadryad.org/stash/share/fne1XRX1SV-LAMM-JovRfdLRIGDfFwyfGp_Gwcssln4).

## Author contributions

GY analyzed the data and wrote the manuscript. LH, GY, and YS conducted data compilation and treatment, data analyzed, and reviewed the manuscript. All authors contributed to the article and approved the submitted version.

## Funding

This study was supported by the National Natural Science Foundation of China (No. 41977420) and Key Research and Development Program of Ningxia Hui Autonomous Region (No. 2021BEG02009).

## Conflict of interest

The authors declare that the research was conducted in the absence of any commercial or financial relationships that could be construed as a potential conflict of interest.

## Publisher's note

All claims expressed in this article are solely those of the authors and do not necessarily represent those of their affiliated organizations, or those of the publisher, the editors and the reviewers. Any product that may be evaluated in this article, or claim that may be made by its manufacturer, is not guaranteed or endorsed by the publisher.
