# Supplementary material for: Magnitude and determinants of plant root hydraulic redistribution: A global synthesis analysis
Source: Front Plant Sci. 2022 Jul 22;13:918585. doi: 10.3389/fpls.2022.918585 (PMC9355616; doi:10.3389/fpls.2022.918585)
Supplement: Supplementary file 2 [file Data_Sheet_2.doc]

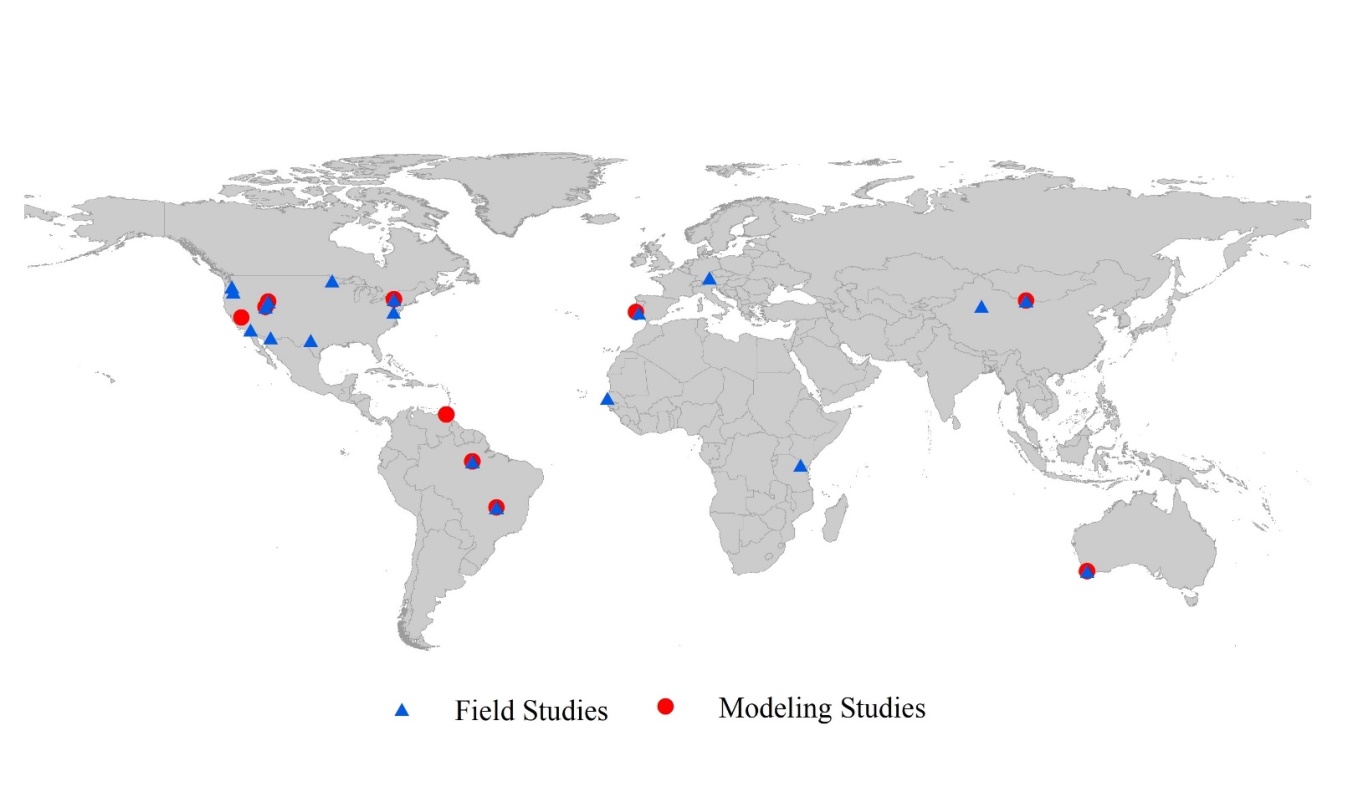


**Figure S1** Location map of 47 HR study sites included in the integrated analysis, field studies (blue triangles, n=35), modelling studies (red circles, n=12)


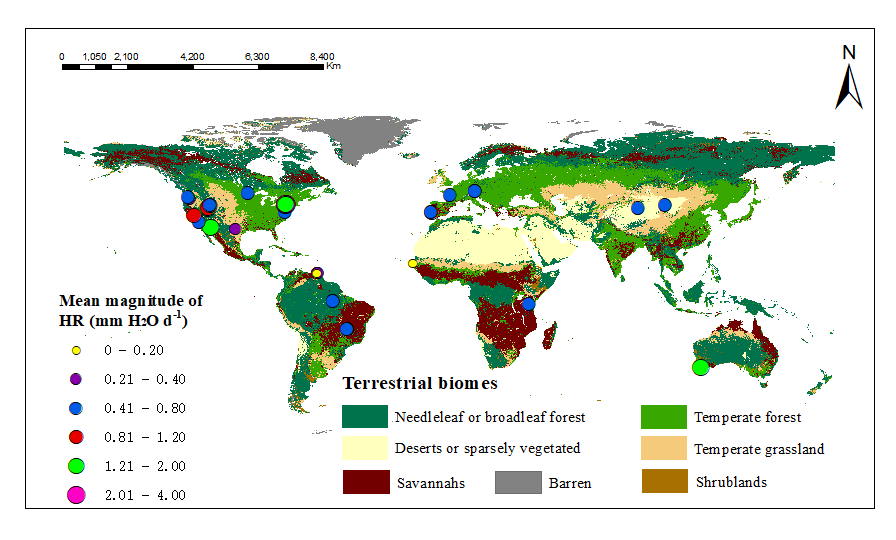


**Figure S2**: Average distribution range of HR in 47 empirical studies; the size of the dot represents the magnitude of HR. The global database terrestrial biomes from The Nature Conservancy. http://www.nature.org.Map ArcMap10.6. [http://www.nature.org.Map](http://www.nature.org.Map/)
